# Evaluation of different Diagnostic Modalities for Diagnosis of Dental Caries: An *in vivo* Study

**DOI:** 10.5005/jp-journals-10005-1385

**Published:** 2016-12-05

**Authors:** Iram Zaidi, Rani Somani, Shipra Jaidka, Muhamad Nishad, Shikha Singh, Divya Tomar

**Affiliations:** 1 Reader, Department of Pedodontics and Preventive Dentistry, SBB Dental College, Ghaziabad, Uttar Pradesh, India; 2 Professor and Head, Department of Pedodontics and Preventive Dentistry, DJ College of Dental Sciences and Research, Ghaziabad, Uttar Pradesh, India; 3 Professor, Department of Pedodontics and Preventive Dentistry, DJ College of Dental Sciences and Research, Ghaziabad, Uttar Pradesh, India; 4 Professor and Head, Department of Pedodontics and Preventive Dentistry, SBB Dental College, Ghaziabad, Uttar Pradesh, India; 5 Reader, Department of Pedodontics and Preventive Dentistry, SBB Dental College, Ghaziabad, Uttar Pradesh, India; 6 Senior Lecturer, Department of Pedodontics and Preventive Dentistry, SBB Dental College, Ghaziabad, Uttar Pradesh, India

**Keywords:** Caries detector dye (Kuraray), DIAGNOdent, Intraoral camera, Occlusal lesion, Visual examination.

## Abstract

**Aim:**

The aim of this study was to compare and evaluate the efficacy of different diagnostic aids for diagnosis of dental caries and to compare the validity in terms of sensitivity and specificity of all four diagnostic modalities for diagnosis of caries.

**Materials and methods:**

Occlusal surfaces of 100 primary and permanent molars were examined using the four diagnostic systems (visual, intraoral camera, DIAGNOdent, and DIAGNOdent with dye). These results were compared with operative intervention gold standard. Sensitivity and specificity were calculated for each diagnostic system for both enamel and dentin caries. Interrater agreement was calculated for each diagnostic system using kappa statistics.

**Results:**

For both enamel and dentin caries, the highest sensitivity values were provided by DIAGNOdent (0.91 and 0.72) and lowest for visual examination on wet surface (0.60 and 0.50). For both enamel and dentin caries, the specificity was found to be highest for intraoral camera on dry surface and lowest for visual examination. The DIAGNOdent gave the highest value of interrater agreement (kappa), i.e., 0.816 as compared with 0.03 for visual examination.

**Conclusion:**

The study clearly demonstrated that DIAGNO-dent was the most accurate and valid system tested for the detection of occlusal caries. It has the advantage of quantifying the mineral content, helping to improve the diagnostic efficacy and treatment and accurate assessment of fissures where the visual examination alone is not adequate, thus complementing the traditional dental examination.

**How to cite this article:**

Zaidi I, Somani R, Jaidka S, Nishad M, Singh S, Tomar D. Evaluation of different Diagnostic Modalities for Diagnosis of Dental Caries: An *in vivo* Study. Int J Clin Pediatr Dent 2016;9(4):320-325.

## INTRODUCTION

Preservation of a healthy set of natural teeth along with the maintenance and integrity of the oral tissue is the primary objective of pediatric dentistry. Dental caries is a most common disease of childhood and despite the modern advances in prevention and increase in understanding of the importance of maintaining the natural dentition, many teeth are lost prematurely. This occurs because of delayed diagnosis of caries.^1^ In the new era of pediatric dentistry where the paradigm has shifted to preservation and minimal intervention, the importance of finding and treating decay in its earliest stages is universally acknowledged.^2^

Throughout both ancient and modern history, mankind tended to accept tooth decay as the main cause of tooth loss.^3^ Traditionally, “Seeing is believing” was the approach adopted by Europeans as diagnostic aid, which was modified by Maury in the 19th century with the invention of mouth mirror and probe.^4^ Thus, visual examination has been the mainstay domain in occlusal caries diagnosis at an early stage for years. But it leads to the possibility of extension of lesion or inoculation of the lesion with cariogenic microorganism.^4^

Introduced by Gorden J. Christensen in 1990, intraoral camera is a feasible alternative to a visual oral examination for caries screening as it gives a magnified view (10× magnification) and large range of viewing angles.^5^ However, despite its increased validity in comparison to visual examination, its sensitivity was usually as much lower as 12% for occult caries, i.e., caries beneath macroscopically intact surface.^6^ Therefore, the urge to search for more advanced methods with good sensitivity along with good specificity that led to the development of the concept of fluorescence for detecting initial caries came into existence.^7^

Based on this, Hibst and Paulus^8^ found that red light-induced fluorescence (655 nm) could reveal considerable contrast between sound and carious tooth tissue. Fluorescence was found to be more intense in carious tissue (140 relative fluorescence intensity) as compared with sound tissue (20 relative fluorescence intensity). Thus, DIAGNOdent has been developed for early detection and quantification of demineralization.^9^

Demineralization of tooth surface is usually associated with discolorations, and based on this, Fusiyama^10^ introduced a technique using basic fuchsine red stain to aid in the differentiation of the two layers of carious dentin, i.e., infected and affected dentin having different ultrastructural and chemical characteristics. Various studies have been done using dyes in conjunction with DIAGNOdent for detecting residual caries with high sensitivity and specificity.^11^

As fear and anxiety in children are recognized barriers to receive dental care, it leads to the requirement of early and timely diagnosis of dental caries to avoid operative radical treatment, especially in children, and for that a caries detection method that should capture the whole continuum of the caries process is required. This study was therefore planned to evaluate efficacy of different diagnostic modalities for early diagnosis of dental caries in children.

## MATERIALS AND METHODS

A prospective study was carried out on 100 untreated molars with enamel and/or dentin caries in 48 children ranging between 5 and 14 years of age, reporting at the Department of Pedodontics and Preventive Dentistry, DJ College of Dental Sciences and Research, Ghaziabad, India, after getting approval from the ethical committee of the institute. The guardians of each of the children were fully informed about the details of the study and asked to sign a consent form authorizing their child’s participation in the study.

The sample comprised 36 first deciduous molars, 30 second deciduous molars, and 34 first permanent molars. The inclusion criteria for the study were (1) untreated primary molars or permanent 1st molar cavi-tated with enamel and/or dentin caries should be present in each sample; (2) patients who were fully cooperative as judged by Frankl and Fogel four-point behavior rating scale;^12^ and (3) patients who had all the teeth and surrounding tissues clinically free from any pathological condition other than caries.

The exclusion criteria for the study included (1) patients who were not cooperative during the selection process; (2) patients having teeth with proximal, buccal, lingual surface lesion; (3) patients having occlusal restorations, fissure sealents, hypoplastic pits, advanced degree of fluorosis, frank occlusal cavitation, large carious lesions on smooth and proximal surface; and (4) teeth and surrounding tissues having any pathological condition other than caries.

Before examination of the carious lesion, the occlusal surface of each tooth was cleaned with rotating bristle brush and water. Thereafter, teeth were first inspected visually followed by the examination with intraoral camera, then by DIAGNOdent, and finally by application of dye and examining by DIAGNOdent.

**Fig. 1:**
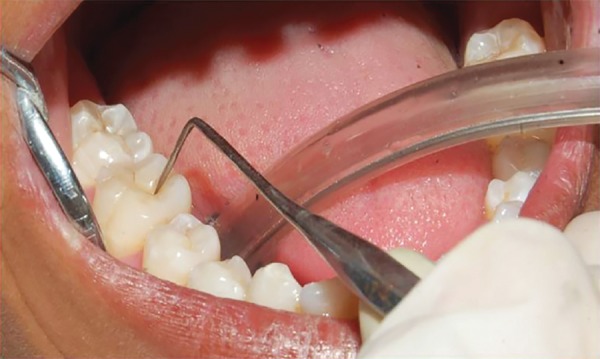
Diagnosis of caries by visual examination

Visual examination was first done on wet surface. The carious surface was examined with mouth mirror under standard dental operating light. The presence or absence of occlusal pit and fissure caries was recorded using the criteria described by Ekstrand^12^ (Table 1). The teeth surface was then dried, and the presence or absence of occlusal pit and fissure caries was recorded using the same criteria used for wet surface (Fig. 1).

**Fig. 2:**
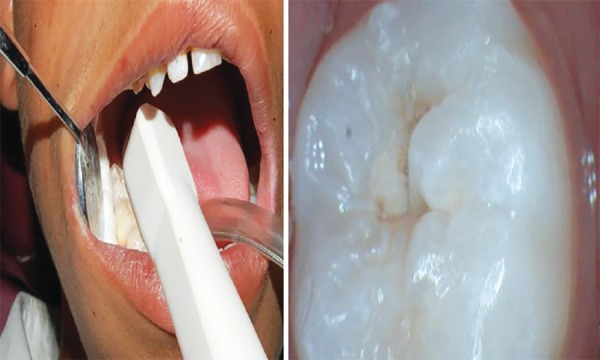
Examination with intraoral camera

After that, the teeth were examined with intraoral camera and mouth mirror. The presence or absence of occlusal pit and fissure caries was recorded on wet surface using Ekstrand criteria.^12^ After moisture control, the carious lesion was again examined with intraoral camera. The presence or absence of occlusal pit and fissure caries was recorded using the same criteria described by Ekstrand^12^ (Fig. 2).

**Table 1:** **Table 1:** Criteria used in visual examination (Ekstrand et al)^12^

*Score*		*Criteria*	
V0		No or slight change in enamel translucency after prolonged air drying (≥5 sec)	
V1		Opacity hardly visible on the wet surface, but distinctly visible after drying.	
V2		Opacity distinctly visible without air drying	
V3		Localized enamel breakdown in opaque or discolored enamel and/or gray discoloration from the underlying dentin	
V4		Cavitation in opaque or discolored enamel exposing the dentin	

**Fig. 3:**
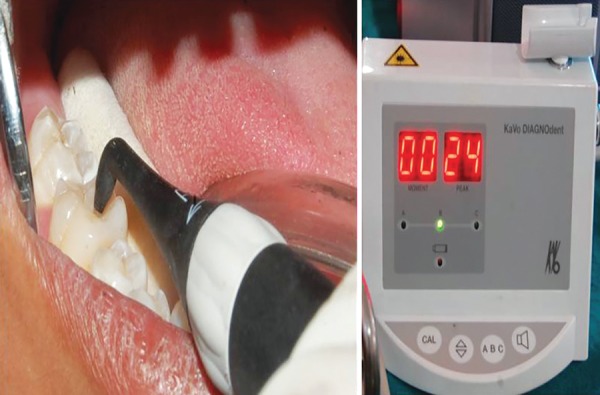
Diagnosis with DIAGNOdent

**Fig. 4:**
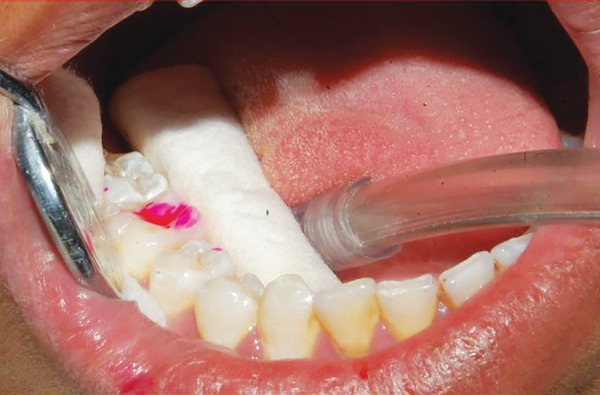
Dye application

**Fig. 5:**
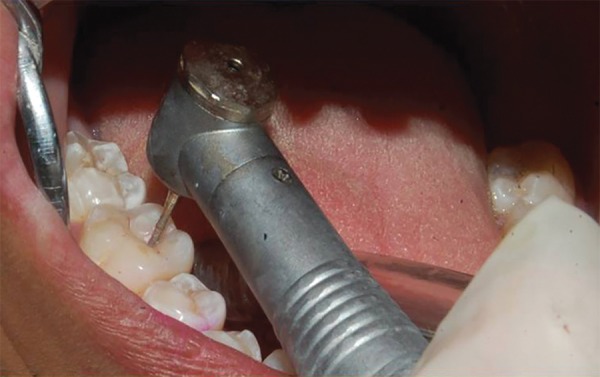
Operative intervention (pit and fissure opening)

**Table 2:** **Table 2:** Criteria used in examination with DIAGNOdent (Lussi et al)^13^

*Score*			*Criteria*	
0-14			No caries	
15-20			Enamel caries	
21-99			Dentinal caries	

After reisolation, the teeth were quantitatively examined by a portable laser fluorescence system (DIAGNOdent, KaVo, Biberach, Germany) (Fig. 3). The instrument was first calibrated against porcelain standard according to the manufacturer’s instruction, and then it was recalibrated after every 10th tooth to minimize calibration drift. The carious tooth was evaluated using probe tip A (used for detection of caries on occlusal surface). The probe tip was placed perpendicularly on the carious surface and was slowly rocked in a pendulous motion to examine the adjacent periphery of the carious surface at various angles, and the maximum value (peak value) was recorded. This peak value was then compared with the DIAGNOdent value based on criteria given by Lussi et al^12^ (Table 2).

Finally, the presence or absence of caries was also detected using caries detector dye propylene gycol (Kuraray). After reisolation, the dye was applied to the occlusal surface using a small applicator (Fig. 4). Dye was then removed after 10 seconds, and then tooth was examined using DIAGNOdent according to the manufacturer’s instructions. The readings of peak value were then recorded according to the criteria given by Lussi et al.^13^

Operative intervention (pit and fissure opening) was done after interpretation of values of all four diagnostic methods (Fig. 5). Out of 100 teeth examined, 8 teeth had a visual score V0 and V1 (Ekstrand criteria) and DIAGNO-dent value less than 15 (Lussi criteria), thus indicating that no caries was present. Thus, remaining 92 occlusal carious molars were opened with an air rotor handpiece. Penetration depth of carious lesion was estimated visually using the World Health Organization (WHO) periodontal probe.

The greatest extent of caries was classified according to the following score given by Ekstrand et al^14^ (Table 3). The restoration of carious teeth was done according to carious lesion. In the teeth having caries up to outer one third of enamel, enameloplasty was done. All the teeth having depth greater than one third of enamel were restored with glass ionomer cement (N100 3M ESPE Manufacturers, Germany).

The data obtained were statistically analyzed to test the validity of all four methods for the lesions in enamel and dentin separately in terms of sensitivity (proportion of carious lesion identified correctly) and specificity (proportion of sound sites identified correctly). The interrater agreement (kappa) was also observed for all four modalities to evaluate the agreement of diagnostic modalities with respect to operative intervention.

**Table 3:** **Table 3:** Criteria used in examination after pit and fissure opening (Ekstrand et al)^14^

*Score*			*Criteria*	
D0			No caries	
D1-D2			Enamel caries	
D3			Caries up to half of dentin	
D4			Caries beyond half of dentin	

## RESULTS

After operative intervention (used as a gold standard), it was found that out of 100 teeth, 8 had no caries (score D0), 20 teeth had enamel caries (score D1, D2), and 72 had caries extending up to dentin (score D3), according to Ekstrand et al^14^ criteria.

**Graph 1:**
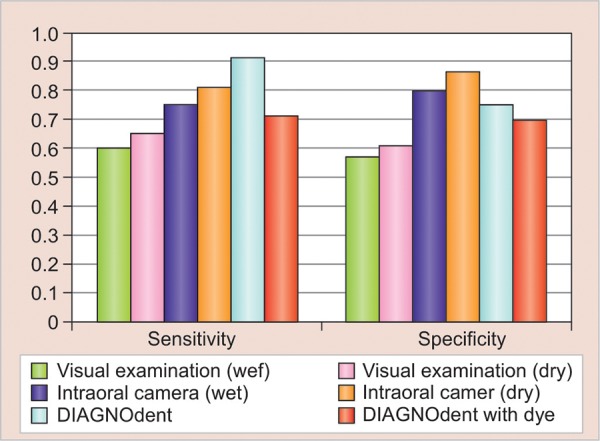
The comparison of sensitivity and specificity of all four tests for enamel caries

The comparison of sensitivity and specificity values for all four methods for detection of enamel and dentin caries has been shown in Table 4. For enamel caries, the sensitivity was found to be highest for DIAGNOdent, i.e., 0.91, and lowest for visual examination (wet), i.e., 0.60, and specificity was found to be highest for intraoral camera (dry), i.e., 0.86, and lowest for visual examination (wet), i.e., 0.57 (Graph 1).

**Graph 2:**
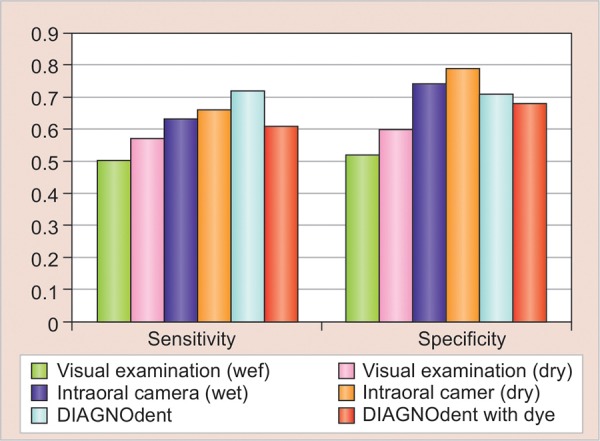
The comparison of sensitivity and specificity of all four tests for dentin caries

For dentin caries, the sensitivity was found to be highest for DIAGNOdent, i.e., 0.72, and lowest for visual examination (wet), i.e., 0.50, while specificity was found to be highest for intraoral camera (dry), i.e., 0.79, and lowest for visual examination (wet), i.e., 0.52. Thus, it was observed that out of the four methods, DIAGNO-dent is the most sensitive and intraoral camera (dry) is the most specific in detecting enamel and dentin caries (Table 4; Graph 2).

**Graph 3:**
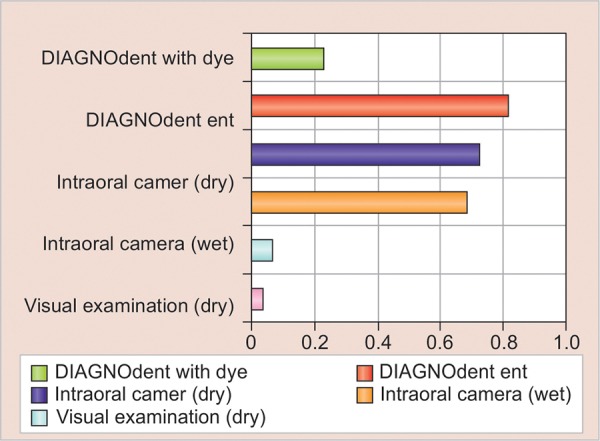
The comparison of kappa of all four tests

The interrater agreement (kappa) was observed for all four modalities to evaluate the agreement of diagnostic modalities with respect to operative intervention. The value of kappa was found to be highest for DIAGNOdent, i.e., 0.816, which signifies almost perfect agreement with operative intervention, and was lowest for visual examination (wet), i.e., 0.03, which denotes slight agreement with operative intervention (Table 5; Graph 3).

**Table 4:** **Table 4:** Sensitivity and specificity of each diagnostic method for enamel (D1-D2) and dentin (D3-D4) caries

		*Enamel caries*				*Dentin caries*			
*Diagnostic* *method used*		*Sensitivity*		*Specificity*		*Sensitivity*		*Specificity*	
Visual									
examination									
Wet		0.60		0.57		0.50		0.52	
Dry		0.65		0.61		0.57		0.60	
Intraoral		0.75		0.80		0.63		0.74	
camera									
Wet		0.81		0.86		0.66		0.79	
Dry									
DIAGNOdent		0.91		0.75		0.72		0.71	
DIAGNOdent		0.71		0.70		0.61		0.68	
with dye									

## DISCUSSION

The total health handicap due to dental caries is staggering and has become a dichotomous disease, especially in children. With an age of specialization and compart-mentalization of knowledge, there has been a decline in prevalence of caries, but it still remains a vestigial remnant of bygone times.^15^

Caries lesions occur on a continuous scale of tissue damage, from subclinical surface changes to macroscopic cavities reaching the pulp, and if detected at an early stage, they can be reversed or remineralized. A diagnostic system should have the advantage of objectively recording non-cavitated stages of carious lesion. The importance of early detection of caries activity is emphasized by the fact that an incipient lesion, which is amenable to remineralization, can be arrested, reversed, or restored with minimal invasion.^16^

For both clinical and epidemiological studies, it is a fundamental premise that diagnostic methods should provide consistent and standardized expressions of the condition in question. This premise places emphasis on the issue of reproducibility and validity of the diagnostic methods available.^17^

Similarly, an increase in sensitivity will be accompanied by a decrease in specificity (increase in the false-positive diagnosis). Considering that a rise in the false-positive proportion can be dangerous as it can lead to overtreatment, a technique that offers high specificity even at the expense of a slight reduction in sensitivity seems to be more appropriate.^18^ Therefore, the methods in this study were evaluated in terms of their validity, i.e., sensitivity and specificity.

**Table 5:** **Table 5:** Measurement of interrater agreement (kappa statistics) of all four diagnostic tests

		*Visual examination*				*Intraoral camera*							
		*Wet*		*Dry*		*Wet*		*Dry*		*DIAGNOdent*		*DIAGNOdent* *with dye*	
Measure of agreement kappa		0.03		0.06		0.680		0.720		0.816		0.524	

In this study, among all diagnostic methods, higher value of sensitivity was found to be for DIAGNOdent, i.e., 0.91, and lowest for visual examination (wet), i.e., 0.60. The reason for increased sensitivity in the present study can be attributed to the fact that DIAGNOdent quantifies the mineral loss as it picks up fluorescence from the slopes of the fissure walls, where the caries process is believed to start and thus lead to early detection of caries.

The present study has demonstrated that the DIAG-NOdent device is capable of obtaining high sensitivity on the occlusal sites of teeth with macroscopically intact surfaces, and it could be used as an aid for longitudinal control of caries as well as to observe outcome of preventive treatment. The performance was similar to that found in an *in vitro* study in deciduous teeth as well as in *in vitro* and *in vivo* studies in permanent teeth.^17^

The possible explanation for less efficacy and validity of visual examination in the present study can be attributed to the fact that, in visual examination, many lesions are left undetected due to the macroscopically intact surface (hidden caries) or wrongly diagnosed as enamel caries, thus allowing the dentinal lesions to progress unchecked. In addition, dental caries is a dynamic process in which early lesions undergo demineralization before being expressed clinically, thus being missed visually.^19^

While evaluating a diagnostic method, it is also important to test whether it provides a reliable and minimal diagnostic variability between measurements to assure consistency and reproducibility along time.^17^ Therefore, to evaluate agreement of diagnostic modalities with respect to operative intervention, the results in this study were also assessed in terms of intrarater agreement (kappa) of diagnostic methods.

The value of kappa was found to be highest for DIAGNOdent, i.e., 0.816, which signifies almost perfect agreement with operative intervention, and was lowest for visual examination (wet), i.e., 0.03, which denotes slight agreement with operative intervention. Thus, the results revealed that DIAGNOdent is a better diagnostic method than the others.

The results were in accordance with the study conducted by Pinheiro et al.^20^ They assessed the accuracy of laser fluorescence (DIAGNOdent) for diagnosis of occlu-sal caries in permanent teeth and found that kappa value is 0.89 for DIAGNOdent and concluded that in routine dental check-up of children, DIAGNOdent appears to be a useful adjunct.^20^

In another study, Rodrigues et al^21^ compared the performance of DIAGNOdent with visual examination for occlusal caries detection and found that DIAGNOdent showed higher sensitivity and lower specificity than did visual examination, and kappa values varied from good to excellent for DIAGNOdent but from poor to good for visual examination.

It was also observed that, in the present study, the use of dye with DIAGNOdent did not improve the result as the sensitivity and specificity in this method were found to be inferior than DIAGNOdent alone. Similarly, the kappa value (0.524) was also lesser than the same for DIAGNOdent (0.816) and intraoral camera methods (wet - 0.620, dry - 0.720), indicating no additional advantage of using dye with DIAGNOdent.

## CONCLUSION

DIAGNOdent is a valid method as it has the advantage of quantifying the mineral content, helping to improve the diagnostic efficacy. The results of the study and inferences drawn show that DIAGNOdent is superior to the currently available methods for detection of initial caries and there is no additional advantage of using dye with DIAGNOdent. Thus, it is conceivable that fluorescence-assisted diagnosis may improve caries diagnosis in future.
